# Validation of a wearable cuff-less wristwatch-type blood pressure monitoring device

**DOI:** 10.1038/s41598-020-75892-y

**Published:** 2020-11-04

**Authors:** Joon Ho Moon, Myung-Kyun Kang, Chang-Eun Choi, Jeonghee Min, Hae-Young Lee, Soo Lim

**Affiliations:** 1 Department of Internal Medicine, Seoul National University College of Medicine, Seoul, South Korea; 2 Department of Internal Medicine, Seoul National University Hospital, Seoul, South Korea; 3 InBody Co., Ltd, Seoul, South Korea; 4 Department of Internal Medicine, Seoul National University Bundang Hospital, Seongnam, South Korea

**Keywords:** Cardiology, Health care, Hypertension

## Abstract

Ambulatory blood pressure (BP) monitoring is recommended to improve the management of hypertension. Here, we investigated the accuracy of BP estimated using a wearable cuff-less device, InBodyWATCH, compared with BP measured using a manual sphygmomanometer. Thirty-five adults were enrolled (age 57.1 ± 17.9 years). The BP was estimated using InBodyWATCH with an individualized estimation based on a neural network model. Three paired sets of BPs from the two devices were compared using correlation analysis and Bland–Altman plots (*n* = 105 paired BP readings). The correlations for both systolic and diastolic BP (SBP and DBP) between the two devices were high (*r* = 0.964 and 0.939, both *P* < 0.001). The mean difference was 2.2 ± 6.1 mmHg for SBP and −0.2 ± 4.2 mmHg for DBP; these were not significant (*P* = 0.472 for SBP and *P* = 0.880 for DBP). The proportions of estimated SBP/DBP obtained from the InBodyWATCH within ± 5 mmHg of manual SBP/DBP were 71.4%/83.8%; within ± 10 mmHg they were 86.7%/98.1%; and within ± 15 mmHg they were 97.1%/99.0%. The estimated BP from this wearable cuff-less device correlated highly with the manual BP and showed good accuracy, suggesting its potential to be used in ambulatory BP monitoring.

## Introduction

Blood pressure (BP) is a vital sign that can be used to indicate the risk for atherosclerotic cardiovascular disease when elevated^1,2^. High blood pressure or hypertension is one of the most prevalent chronic diseases that threaten human health worldwide, regardless of age, gender, body mass index, ethnicity, or economic condition^3–6^. Recent guidelines for hypertension from the American College of Cardiology (ACC), the American Heart Association (AHA) and the European Society of Hypertension (ESH) recommend measuring out-of-office BP including ambulatory blood pressure monitoring (ABPM) and home BP in addition to office BP^7,8^. However, ABPM is not frequently used in real-world practice, mainly because of the many disturbances that can occur during measurement, such as the large size of devices, inconvenience in application and pain or a sense of oppression from frequent measurements^9,10^.

Currently, along with the evolution of the Internet of Things and artificial intelligence, research and developments on wearable devices that can measure BP ubiquitously are actively being conducted in industries. These wearable cuff-less devices estimate BP from signals generated by the human body. Therefore, the validation of BP values from these devices is mandatory if they are to be employed in clinical practice.

BPs are commonly estimated mathematically from pulse wave velocity (PWV) and pulse transit time (PTT)^11–14^. PTT, which has an inverse correlation with BP and PWV, can be calculated as the time interval between the peak of the R wave of an electrocardiograph (ECG) and the peak of the derivative of photoplethysmography (PPG). PWVs can be measured from different peripheral sites, such as brachial–ankle PWV (baPWV), carotid–radial artery PWV (crPWV) and femoral–ankle PWV (faPWV). The crPWV is associated with systolic BP (SBP), diastolic BP (DBP) and total peripheral resistance, suggesting a potential use of peripheral PWV to estimate BP and assess arterial stiffness and cardiovascular risk^15,16^.

Wristwatch-type devices are considered the most comfortable to be applied throughout the day so are under active development. A wristwatch-type BP monitoring device was developed based on an oscillometric method using a cuff with a limited wrist circumference range (medium 16.0–19.0 cm; large 18.0–21.5 cm; Heartguide; Omron Healthcare, Kyoto, Japan)^17^. By contrast, cuff-less devices acquire PPG information from the wrist, which only requires an interface between the PPG sensor and the wrist surface, thereby providing potential for convenient measurement. It measures volumetric changes to capillaries on the wrist surface instead of measuring blood flow into the radial artery directly. To compensate for the limitations in accuracy for signals measured at the wrist, attempts to incorporate machine learning including deep neural networks have been made^18^. In the same vein, the Institute of Electrical and Electronics Engineers standard (IEEE Std) 1708 for wearable cuff-less BP measuring devices mandates a calibration process in a validation process to estimate unknown parameters^19^. Recently, a BP-monitoring application was launched based on PPG signals, which estimates BP based on a calibration against manual BP measurements (Galaxy Watch Active 2; Samsung Electronics, Suwon, South Korea).

Here, we investigated the accuracy and correlation between PPG-based BPs measured with a wearable cuff-less device (InBodyWATCH; InBody Co., Ltd., Seoul, South Korea) and BPs measured with a manual sphygmomanometer. We validated an individualized calibration algorithm for the device using a neural network approach.

## Methods

### Study design

In this study, 40 adults who visited Seoul National University Bundang Hospital (SNUBH) outpatient clinic were screened and 35 (17 men, 18 women) were enrolled based on the selection criteria as below. All subjects participated voluntarily, and informed consent was obtained from each of them. This study was approved by the Institutional Review Board of SNUBH (IRB# B-1811-505-001) and was conducted according to the Declaration of Helsinki (2013).

### Enrolment criteria

Inclusion criteria were as follows: men and women aged over 20 years and who had normal sinus rhythm. Exclusion criteria were as follows: those who had serious orthopaedic problems such as fractures, malformations or severe osteoarthritis in the upper arms; who had received an intravascular injection in the arm within 2 days; subjects who could not sit and rise unassisted; who had Parkinson’s disease or tic disorder; who had a history of fainting myocardial infarction, heart failure, severe liver failure or kidney failure; those who had received major surgery within 3 months; subjects who had psychological problems (e.g., schizophrenia, epilepsy, alcoholism, drug addiction or anorexia nervosa); those who were cardiac pacemaker-dependent or had severe carotid stenosis; those who exercised severely or consumed coffee within 1 h on the day of measurement; or those who refused to participate in this clinical study. Pregnant women were also excluded.

### BP measurement

Participants were seated and guided to wear the wearable BP monitoring device on the left wrist (InBodyWATCH; InBody Co., Ltd.) (Supplementary Fig. S1). Manual BPs were measured in the left upper arm using a manual sphygmomanometer (BPBIO220, InBody Co., Ltd.) by two independent researchers. If there was a difference in the measured BP over 4 mmHg between the two investigators, the BP was measured again.

**Figure 1:**
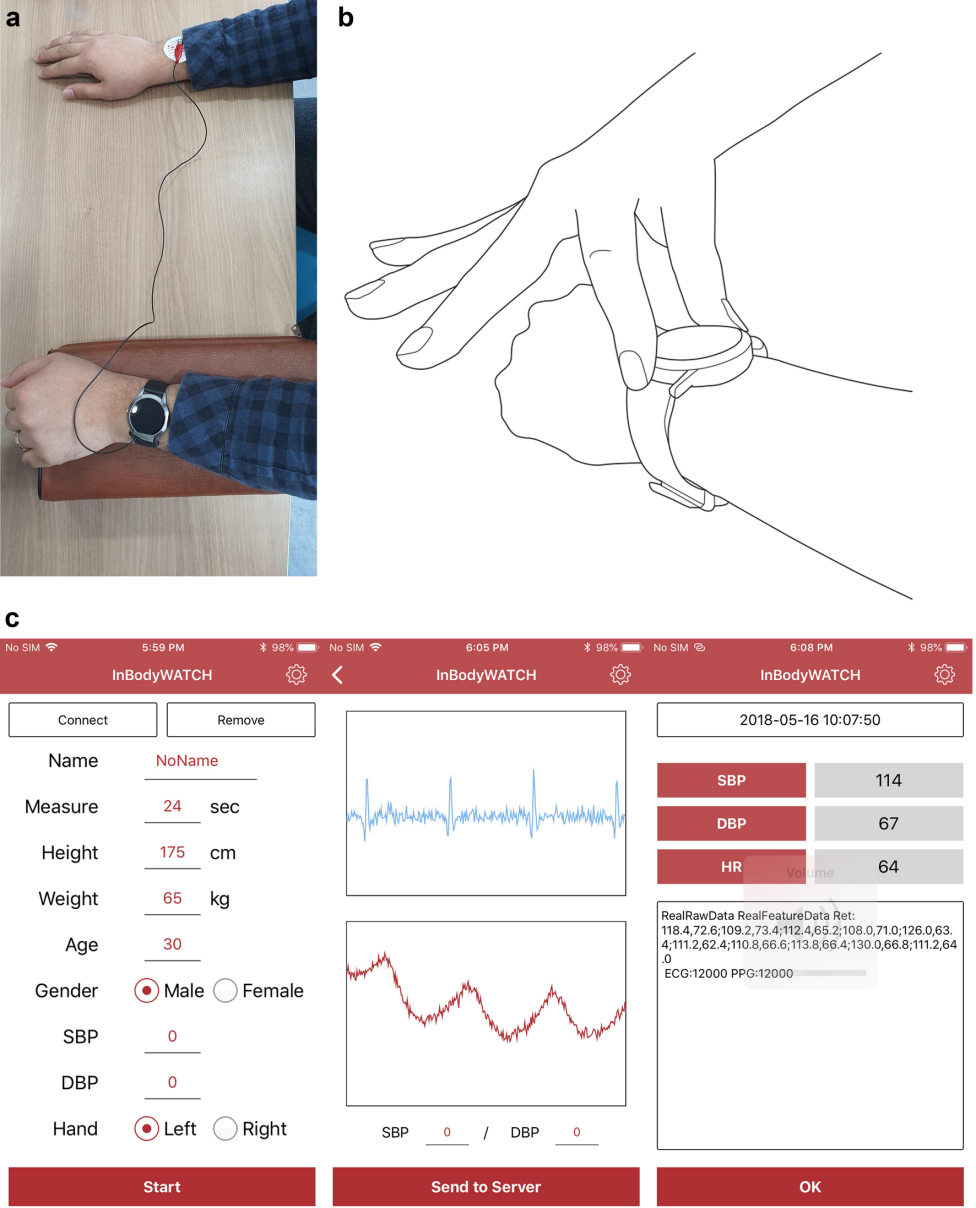
Wearable device (InBodyWATCH) and its application. (**a**) An adhesive electrode was attached to the right wrist to acquire ECG signals. (**b**) ECG signals was collected by putting a right-hand finger on the wristwatch. (**c**) An example of data display generated from the wearable device in the iOS system.

The new device automatically collects PPG and ECG signals. These were processed to generate BP estimates through a pretrained BP model based on a neural network model described below. The PPG signal was obtained at the left wrist with a PPG module using a green light-emitting diode (LED) and photodiode. For this study, an adhesive electrode was attached to the right wrist and connected to the upper electrodes of the monitoring device (Fig. 1a). Signals can also be obtained by putting the right finger on the device at the left wrist (Fig. 1b). In a preliminary study, the proportions of estimated BP from the finger-on-watch/adhesive electrode with differences less than 5/10/15 mmHg compared with manual BP measurements were: for SBP, 60.0%/62.2% (± 5 mmHg); 95.6%/91.1% (± 10 mmHg); 97.8%/100% (± 15 mmHg); for DBP, 73.3%/71.1% (± 5 mmHg); 100%/91.1% (± 10 mmHg); 100%/100% (± 15 mmHg). The mean error and standard deviation compared with manual BP measurement was 2.4 ± 5.6 mmHg (SBP) and –0.7 ± 4.4 mmHg (DBP) for the finger-on-watch method and 0.6 ± 6.3 mmHg (SBP) and –3.0 ± 5.0 mmHg (DBP) for the adhesive electrode. Given that the proportions of BP-in-range and standard deviation were similar between the two methods, we expect that the accuracy is also similar.

The PPG and ECG signals were collected for 24 s and sent to an Android smartphone via Bluetooth low energy communication. An application provided by the manufacturer was installed in the phone that was used to transfer signals from the BP monitoring device to the server and to receive estimated BP from the server (Fig. 1c). Signals with personal information including age, gender, body weight and height were transferred from the database of the smartphone of the participant to the server. The server processed the signals and personal characteristics to generate features and estimate BP with its in-built model.

To configure unknown variables that might be missing from PPG and ECG signals, the BP devices were calibrated individually for each participant. This was done by matching three estimated BP values from the new BP monitoring device (Watch C1, C2, and C3) with three reference manual BP measurements (Manual C1, C2, and C3) (Supplementary Fig. S2).

To obtain the primary data for analyses, SBP and DBP were measured 5–10 min after calibration with the manual sphygmomanometer and the InBodyWATCH four times and three times, respectively, starting with the manual sphygmomanometer (Supplementary Fig. S2). Estimated BP from the InBodyWATCH was compared with the closest values of the previous and next manual BP measurements to calculate correlation and accuracy. These seven sequential measurements followed the ESH International Protocol revision of the 2010 Protocol, which corresponds to BP1 to BP7^20^. The schematic of BP estimation is summarized in Supplementary Fig. S3.

### BP estimation modelling

The BP estimation model has two parts: (1) a general BP estimation model and (2) calibration. Prior to this study, a general BP estimation model based on a neural-network model was trained on a dataset by the manufacturer (n = 294). The general BP estimation model generated by the manufacturer was constant for this study. Calibration parameters were calculated for each participant during calibration measurements.

1. General BP estimation model

The general BP estimation model was built in two steps: i) feature extraction, and ii) model training based on a neural network. Features were extracted from the ECG/PPG data obtained from InBodyWATCH with the following processes: removing noise (denoising), beat separating, signal processing, and signal quality checking. First, PPG and ECG signals were denoised with bandpass filters (0-phase delay Butterworth type, PPG: 0.8–11 Hz, ECG: 5–30 Hz). Then, ECG-PPG signals were separated on each beat to obtain training data and to exclude beats with poor signal quality. One 24-s measurement extracted 10 beat data, which were chosen by signal quality checking. Features including PTT, a spectrum of PPG, and additional features from the signals were extracted for each beat^21^. At the final stage of feature extraction, spatial correlations of PPG signals from each beat were calculated. If these correlations were below a reference range, the whole measurement was excluded from the training dataset. This signal quality check process was conducted to avoid poor and inconsistently shaped PPG signals caused by unstable contact between the PPG sensor and the wrist. This signal quality checking process was also adopted for the primary data analysis to prevent BP estimations based on low quality signals (Supplementary Fig. S3).

Before training the general BP estimation model, outliers in the dataset were excluded. Outliers were detected in two steps. First, means and standard deviations of the features listed above were calculated. Data were excluded from the dataset if any of the selected features of the data exceeded a 3-sigma (standard deviation [SD]) range. Second, the suboptimal auto-encoder and decoder were trained against the features^22^. The mean squared error of the auto-encoder for each data point was defined by averaging the squared sums of differences between features before encoding, and reconstructed features from the auto-encoder. If the mean squared error of data exceeded the 3-sigma range, that point was excluded from the dataset based on the assumption that the error arose from an abnormal feature in the data. Then, the dataset was divided into training and test sets where the model was trained and the parameters such as number of layers, activation function, and number of nodes were optimized to minimize any loss of validation for any given dataset.

This general BP estimation model was trained and chosen using the following processes. We chose a fully connected neural network in which every node of one layer was connected to every node of the next layer. The output layer had two nodes: SBP and DBP. Therefore, the SBP and DBP model can be considered as a result of two linear regression models sharing the same features as the final hidden layer generated from previous neural network layers. Based on this, the numbers of layers and the numbers of nodes per layer were changed and trained repeatedly to find an optimal model based on cross-validation. During the training process, batch normalization was applied to each layer to enhance the stability of learning and to prevent overfitting^23^.

(2) Calibration

For calibration, it was not realistic to train additional machine learning models using limited sets of manual and estimated BPs for individual BP estimations. Instead, we added correction terms between final hidden and output layers of a general BP estimation model. Correction terms were determined from ≥ 3 pairs of manual and InBodyWATCH BP measurements. The schematic for the calibration process is illustrated in Supplementary Fig. S4.

### Wearable cuff-less BP monitoring device

The InBodyWATCH is a wearable activity tracker with a diameter of 38.0 mm, 11.6 mm thickness and weighing 31.2 g. It provides activity tracking functions such as step counts, sleep tracking, and heart rate measurement altogether with Bluetooth v. 4.0 functions, a 128 × 128-pixel monochrome organic light-emitting diode (OLED) display, and a 3.7 V direct current (DC) 140 mAh battery (Supplementary Fig. S1). Two bottom electrodes and one top electrode are used to obtain ECG signals by measuring the voltage difference between the left wrist (bottom electrodes) and the right arm (top electrode). A PPG sensor (with green LED) placed on the base is used to measure volumetric changes in blood vessels by alternating current (AC) modulation in reflected LED light, as blood behaves as an absorber of LED light.

### Baseline examination

At baseline, body composition was analysed using the bioelectrical impedance analysis method with a body composition analyser (InBody 770, InBody Co., Ltd.), which has been used commonly in other studies^24^. Information about age, gender, medical history including any hypertension, and medication lists was collected. Vital signs, body weight, height, arm circumference, wrist circumference and arm length were measured. The second BP measurement during calibration was used as the baseline (Supplementary Table S1). A complete blood cell count analysis including white blood cell, haemoglobin, haematocrit and platelet counts was performed using an XE-2100 device (Sysmex, Kobe, Japan). Plasma glucose concentration was measured using the glucose oxidase method (747 Clinical Chemistry Analyzer; Hitachi, Tokyo, Japan). Glycated haemoglobin (HbA1c) was measured using a Bio-Rad Variant II Turbo High Performance Liquid Chromatography Analyzer (Bio-Rad, Hercules, CA, USA). Alanine and aspartate aminotransferase (ALT and AST, respectively) levels were measured using the Nicotinamide Adenine Dinucleotide Coenzyme-Ultraviolet method, and serum creatinine was measured by Jaffe’s kinetic method using a Hitachi 747 Clinical Chemistry Analyzer (Hitachi, Tokyo, Japan). Serum total cholesterol, triglycerides, high-density lipoprotein (HDL)-cholesterol, and low-density lipoprotein (LDL)-cholesterol levels were measured using a 747 Clinical Chemistry Analyzer (Hitachi).

### Statistical analysis

Differences between mean BP values measured by the manual sphygmomanometer and those calculated by the InBodyWATCH were compared using Student’s *t* test. Pearson’s correlation coefficient (*r*) was calculated and *r* > 0.90 was considered to indicate a high correlation. Bland–Altman plots were used to compare differences between techniques, and the ratio of measurements with BP differences of 5 mmHg, 10 mmHg, and 15 mmHg, and the root mean squared error (RMSE) are presented. Data are presented as the mean ± SD. Statistical significance was accepted when *P* < 0.05. Statistical analyses were conducted using the statistical functions in Python SciPy packages (version 1.1.0; available from: https://www.scipy.org/)^25^. Scatter plots and Bland–Altman plots were drawn using Python Matplotlib package (version 3.1.3; available from: https://matplotlib.org/)^26^.

## Results

### Participant characteristics

Forty subjects were screened for this study. Among them, 35 (57.1 ± 17.9 years old, 17 men and 18 women) were included in the analysis, excluding four who did not have the device calibrated and one who did not undertake a baseline body composition analysis. Baseline characteristics of the participants are shown in Table 1. Nine of the participants (26%) had been diagnosed with hypertension; four (11%) had dyslipidaemia, and two (6%) had a history of stroke. Age and BP distributions of the participants are listed in Supplementary Table S1.

**Table 1:** Baseline characteristics of study participants.

Variables	Mean ± SD or *n*
Age (years)	57.1 ± 17.9
Gender (Male/Female)	17/18
Height (cm)	162.2 ± 8.6
Body weight (kg)	67.5 ± 12.3
Body mass index (kg/m^2^)	25.6 ± 3.9
Fasting glucose (mg/dL)	129.0 ± 58.0
HbA1c (%)	6.9 ± 1.5
AST (IU/L)	32.5 ± 17.7
ALT (IU/L)	41.2 ± 36.4
Serum creatinine (mg/dL)	0.9 ± 0.4
Total cholesterol (mg/dL)	171.2 ± 39.0
Triglycerides (mg/dL)	153.3 ± 153.7
HDL-cholesterol (mg/dL)	47.6 ± 11.1
LDL-cholesterol (mg/dL)	103.5 ± 27.9
White blood cell (× 10^3^/μL)	7.1 ± 1.5
Haemoglobin (g/dL)	13.9 ± 1.7
Platelets (× 10^3^/μL)	240.6 ± 51.3
**Body composition analysis**	
Percent body fat (%)	32.2 ± 6.7
Abdominal visceral fat area (cm^2^)	105.7 ± 36.5
**BP measured using a manual sphygmomanometer**	
SBP (mmHg)	129.6 ± 23.6
DBP (mmHg)	69.5 ± 12.9

In all, 35 subjects were included in the study. Data are presented as the mean ± standard deviation (SD). HbA1c, glycated haemoglobin; AST and ALT, aspartate and alanine aminotransferases, respectively; HDL, high-density lipoprotein; LDL, low-density lipoprotein; SBP, systolic blood pressure; DBP, diastolic blood pressure.

### Accuracy and correlation of SBP measured by the new BP monitoring device compared with manual BP measurement

BPs were measured three times using the InBodyWATCH, preceded and followed by manual BP measurement (four times) using a manual sphygmomanometer. The estimated BP from the InBodyWATCH was compared with the nearer values of the previous and next manual BP measurement^20^. In all, 105 paired BP readings from the 35 subjects were included for analysis.

**Figure 2:**
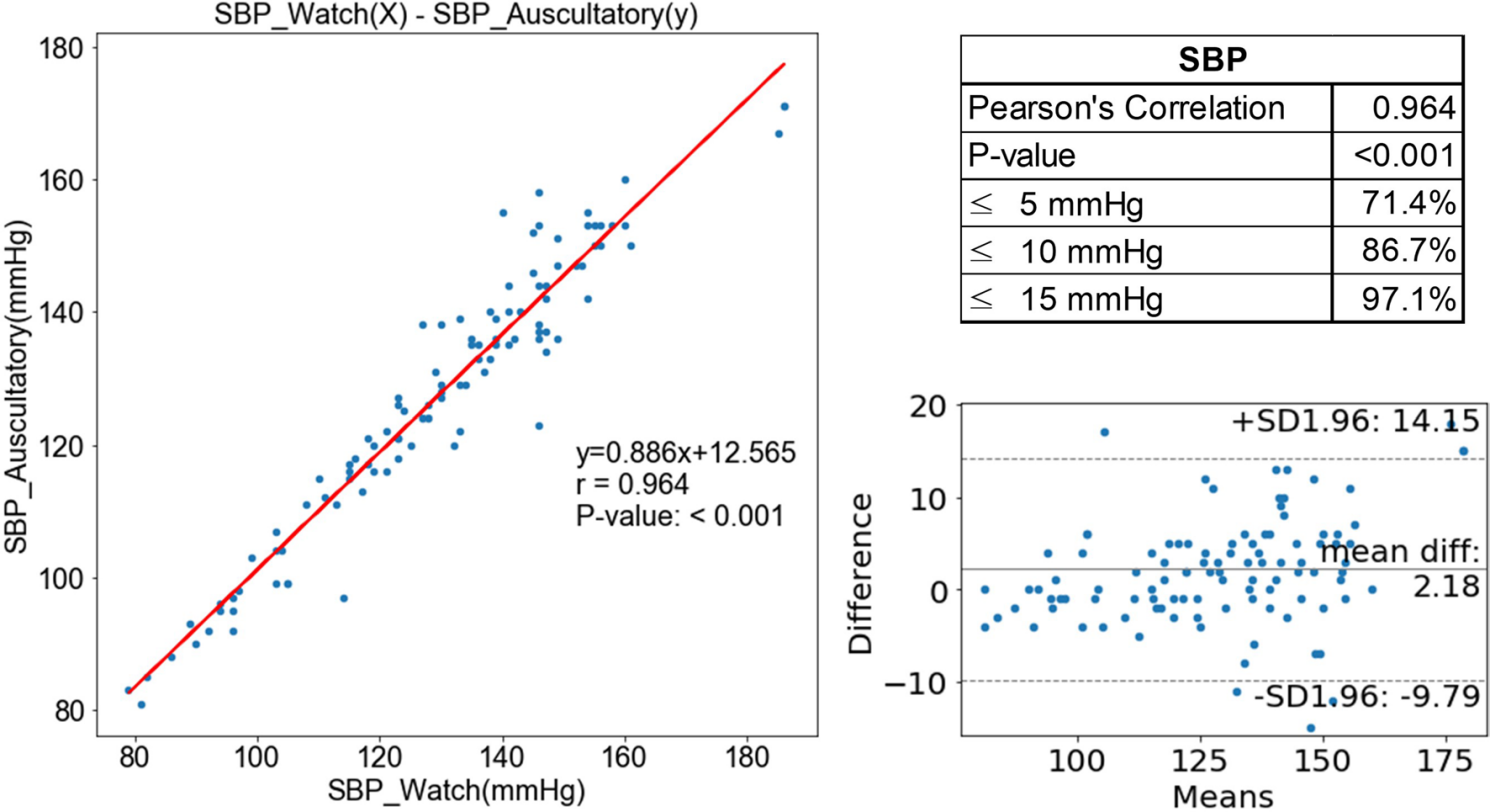
Correlation of the accuracy of systolic blood pressure (SBP) measurements between the InBodyWATCH (SBP_Watch) and a manual sphygmomanometer (SBP_Auscultatory).

The mean SBP values measured by the manual sphygmomanometer and InBodyWATCH were 127.2 ± 20.9 mmHg and 129.4 ± 22.8 mmHg, respectively. The mean difference between the two measures was 2.2 ± 7.3 mmHg; this was not significant (*P* = 0.472) and the correlation between the two values was high (*r* = 0.964, *P* < 0.001) (Fig. 2a). The proportions of paired readings for which the SBP difference was within ± 5 mmHg, ± 10 mmHg, and ± 15 mmHg were 71.4%, 86.7% and 97.1%, respectively (Fig. 2b). The RMSE was 6.5 mmHg.

### Accuracy and correlation of DBP measured by the new BP monitoring device compared with manual BP measurement

**Figure 3:**
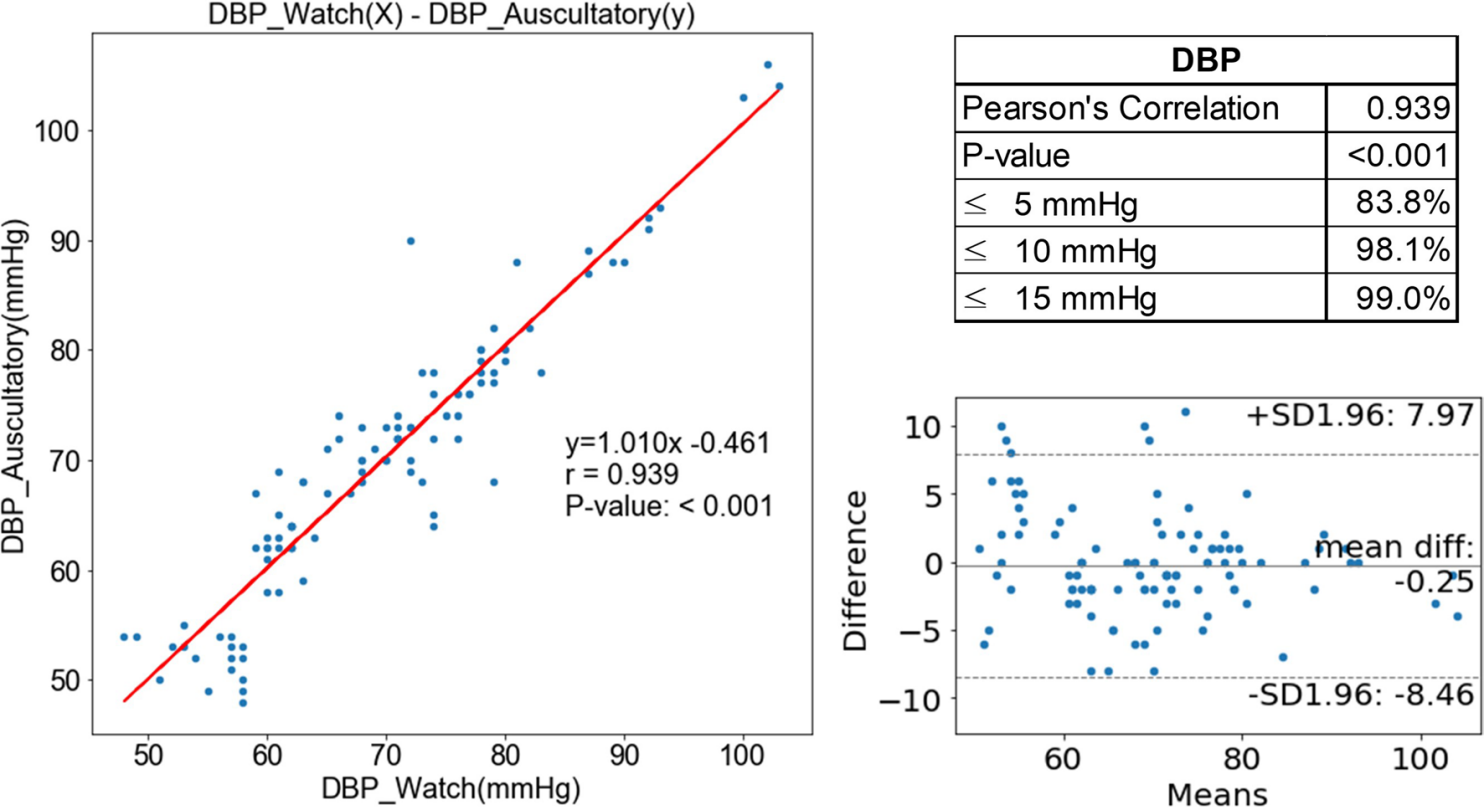
Correlation of the accuracy of diastolic blood pressure (DBP) measurements between the InBodyWATCH (DBP_Watch) and a manual sphygmomanometer (DBP_Auscultatory).

The mean DBP values measured by the manual sphygmomanometer and InBodyWATCH were 69.7 ± 12.3 mmHg and 69.5 ± 11.5 mmHg, respectively. The mean difference between the two measures was −0.2 ± 4.2 mmHg; this was also not significant (*P* = 0.880), and there was a high correlation between the two values (*r* = 0.939, *P* < 0.001) (Fig. 3a). The proportions of paired readings for which the DBP difference was within ± 5 mmHg, ± 10 mmHg, and ± 15 mmHg were 83.8%, 98.1% and 99.0%, respectively (Fig. 3b). The RMSE was 4.2 mmHg.

### Diagnostic accuracy of hypertension using the new BP monitoring device

The accuracy of diagnosing hypertension was determined using the BPs from the manual sphygmomanometer and InBodyWATCH (Table 2). Nine of 105 SBP measurements were discordant for high SBP criteria (> 135 mmHg) and one was discordant for high DBP criteria (> 85 mmHg), suggesting higher accuracy of DBP using the InBodyWATCH. Only one subject was misdiagnosed as having hypertension when this was defined as high SBP/DBP values (> 135/85 mmHg) in three measurements twice or more (accuracy, 97.1%).

**Table 2:** Diagnostic accuracy of hypertension using the new ambulatory blood pressure monitoring (ABPM) device.

SBP 1		SBP 2		SBP 3		DBP 1		DBP 2		DBP 3		Hypertension	
Device	Manual	Device	Manual	Device	Manual	Device	Manual	Device	Manual	Device	Manual	Device	Manual
130*	138	127*	138	133*	139	74	72	70	70	78	79	×	O
146*	123	133	122	132	120	79	68	73	68	72	70	×	×
136*	135	137*	131	134	129	102	106	103	104	100	103	O	O
153	147	146	138	147*	134	57	52	51	50	58	50	O	O
141	144	139	135	136*	133	82	82	79	82	80	79	O	O
141	136	138*	133	130	128	93	93	92	92	92	91	O	O
155	153	160	160	146	153	81	88	87	89	72*	90	O	O

A total of 10 of 210 BP measurements that were discordant for high systolic blood pressure (SBP) or diastolic blood pressure (DBP) (> 135/85 mmHg) between the manual BP and the estimated BP from the ABPM device are marked with an asterisk (*). Hypertension was defined as ≥ 2 high SBP or DBP in three measurements.

## Discussion

In this study, the estimated BPs from the InBodyWATCH had significant correlations with the manual BP values with correlation coefficients *r* > 0.9 and little difference from manually measured BPs using a sphygmomanometer with > 85% of the paired readings within 10 mmHg. The results suggest the accuracy of our individualized calibration algorithm using a neural network model in the estimation of BPs. Given the high accuracy and correlations, the wearable device has shown the potential to become an option for ABPM in a real-world setting.

Hypertension is the most prevalent chronic disease and increases the risk of cardiovascular disease. The advantages of ABPM, including stronger prognostic evidence and avoiding the spurious diagnostic potential of ‘white-coat and masked’ measurements, were reasons for strong recommendation in recent ACC/AHA and ESH guidelines^7,8^.

A higher ABPM independently predicted adverse cardiovascular events even after adjustment for office-based BP measurement in a 5-year follow-up study including ~ 2000 hypertensive subjects^27^. In the Pressioni Arteriose Monitorate e Loro Associazioni study, the predictability of 11-year cardiovascular mortality gradually increased from office to home to ambulatory BP in the general population^28^. Of note, high night-time BP was a strong predictor for adverse cardiovascular events^29^. Thus, continuous ABPM has clinical benefits and now has become essential in the management of hypertension.

The drawbacks of ABPM are its high cost and inconvenience in wearing and carrying a measuring device. Frequent pressure on the upper arm also disturbs the subjects’ activity. In this study, we developed a wearable, wristwatch-type, cuff-less BP measuring device, which can overcome these disadvantages. This totally mobile, radial artery-based device was highly accurate in BP estimation compared with a conventional manual sphygmomanometer.

Wearable devices that were developed previously had either a pressure cuff or an additional sensor other than a wristwatch. Devices that collect signals from the upper arm^30^, ventral side of the wrist^31^, or tethered to a wire^31^ add inconvenience, which can lead to poor compliance. Therefore, a wristwatch system based on ECG and PPG without a cuff was developed to measure BP without inconveniencing the subject^13,17^. Except for Samsung Electronics, major information technology companies have yet to provide BP measurement systems on their health-care wearables, as it is still difficult to achieve accuracy in the estimated BP. Thomas et al. suggested different formulae to calculate BP, but the RMSE between the estimated BP and the reference BP values were 7.83 mmHg for SBP and 5.77 mmHg for DBP with the best fitting formula (RMSE, 6.5 mmHg for SBP and 4.20 mmHg for DBP in our study)^13^. The Omron Heartguide was reported to have differences of 0.8 ± 12.8 mmHg for SBP and 3.2 ± 17.0 mmHg between watch-based BP and ambulatory BP measurements^17^. To our knowledge, no technical validation report of Samsung Galaxy Watch Active 2 (Samsung Electronics) has been published except for a brief report from the Korea Food and Drug Administration; the difference between the manual and the estimated BP was within ± 5 mmHg with a standard deviation of 8 mmHg (2.2 ± 6.1 mmHg for SBP and –0.2 ± 4.2 mmHg for DBP in our previous study)^32^. Here, we employed a neural network model to fit ECG and PPG-based BP optimally to the reference manually measured BP, which enhanced the accuracy and correlation of the device-estimated BP compared with existing wearable BP devices.

### Strengths and limitations

This study had several strengths. First, we included subjects with a diverse range of ages and BPs to comply with ESH criteria, whereas previous studies were limited to healthy young subjects (Supplementary Table S1)^20,33^. We found that the device–server system worked systematically with methodologies using a neural network approach for calibration. Notably, the technology we used in this device is expected to enable ABPM estimation models to be enhanced gradually with the accumulation of data transferred into the server from a variety of users. In addition, the BP monitoring device detects signals from the wrist without pressure and is completely wireless, different from those devices that use pressure cuffs or collect signals from the upper arm or brachial artery, requiring additional devices or steps to measure BP ^17,30^. Further, the device is compact and light: the InBodyWATCH is 38.0 mm in diameter and 11.6 mm thick, weighing 31.2 g; the same parameters for the Omron Heartguide are 48 mm, 14 mm, and 115 g (including the band weight); and for the Samsung Galaxy Watch Active 2, there are 40.0–44.0 mm, 10.9 mm, and 26–42 g. Finally, BP is estimated in less than 30 s without any preparation other than wearing the wristwatch.

There were also several limitations in this study. First, estimation of BP from the wrist region has some intrinsic limitations. Forward blood flow is reflected from the resistant artery and merged with the backward flow. As a result, the BPs measured in the radial artery have a higher SBP value than those measured in the forearm, but we tried to compensate for this error with individual calibration. Second, we used an electrode attached to the right wrist, instead of the right finger being placed on the watch. Third, the device needs to be validated in other conditions including different body positions (e.g., prone) to evaluate whether the calibration fits well in other conditions. Nevertheless, given that management of hypertension is based on the BP measured during resting and seating, our device exhibited high correlations and accuracy in the seated position, well enough to be applied to BP management. Fourth, this research validated the accuracy of InBodyWATCH BP measurements shortly after calibration. Longitudinal validation is needed to estimate the calibration frequency needed for this device: e.g., Galaxy Watch Active 2 instructions recommend users to update calibration within 4 weeks^32^.

### Conclusions

We have developed a totally mobile, radial artery-based BP estimation using a wearable cuff-less wristwatch and validated its accuracy in comparison with a manual sphygmomanometer. Application of such ABPM devices will improve BP control rates in subjects with hypertension. With advances in technology, more precise ABPM is likely to become available and this will yield clinical benefits such as reductions in cardiovascular morbidity and mortality. Recent studies suggest the potential contribution of crPWV to assess arterial stiffness and cardiovascular outcomes^15,16^. Equipped with information on physical activity and body fat, more sophisticated devices that can measure other vital signals, such as pulse rate, ECG, oxygen saturation, and body composition, will help in preventing and treating many chronic diseases such as hypertension and diabetes mellitus more effectively, and will help alter the management of chronic cardiometabolic disorders.

## Supplementary information

Supplementary Information

## Data Availability

All data associated with this study are present in the paper or available from the corresponding author on reasonable request.
