# Supplementary material for: Validation of a wearable cuff-less wristwatch-type blood pressure monitoring device
Source: Sci Rep. 2020 Nov 4;10:19015. doi: 10.1038/s41598-020-75892-y (PMC7642418; doi:10.1038/s41598-020-75892-y)

**Validation of a wearable cuff-less wristwatch-type blood pressure monitoring device**

Running title: A wearable blood pressure monitoring device

Joon Ho Moon^1,2*^, Myung-Kyun Kang^3*^, Chang-Eun Choi^3^, Jeonghee Min^4^, Hae-Young Lee^1,2^ & Soo Lim^1,4^

^1^ Department of Internal Medicine, Seoul National University College of Medicine, Seoul, South Korea

^2^ Department of Internal Medicine, Seoul National University Hospital, Seoul, South Korea

^3^ InBody Co., Ltd., Seoul, South Korea

^4^ Department of Internal Medicine, Seoul National University Bundang Hospital, Seongnam, South Korea

*The first two authors contributed to this study equally.

**Correspondence to Soo Lim, MD, PhD

Professor, Department of Internal Medicine, Seoul National University College of Medicine, Seoul National University Bundang Hospital

82, Gumi-ro 173 Beon-gil, Bundang-gu, Seongnam-city, South Korea (Postal code: 13620)

T: 82-31-787-7035, F: 82-31-787-4051, M: 82-10-9766-2706, [limsoo@snu.ac.kr](mailto:limsoo@snu.ac.kr)

**Supplementary Tables and Figures**

| Supplementary Table S1. Age and blood pressure distributions of participants. | | | |
| --- | --- | --- | --- |
|  |  | Men | Women |
| Age (years) | ≤40 | 1 | 6 |
|  | 41–50 | 3 | 1 |
|  | 51–60 | 3 | 3 |
|  | ≥61 | 10 | 8 |
| SBP (mmHg) | ≤89 | 0 | 2 |
|  | 90–129 | 7 | 8 |
|  | 130–160 | 9 | 6 |
|  | 161–180 | 1 | 1 |
|  | ≥181 | 0 | 1 |
| DBP (mmHg) | ≤39 | 0 | 0 |
|  | 40–79 | 16 | 13 |
|  | 80–100 | 0 | 5 |
|  | 101–130 | 1 | 0 |
|  | ≥131 | 0 | 0 |

SBP, systolic blood pressure; DBP, diastolic blood pressure.

**Supplementary Figure S1.** The wearable cuff-less ambulatory blood pressure measurement device (InBodyWATCH) used in this study.

**
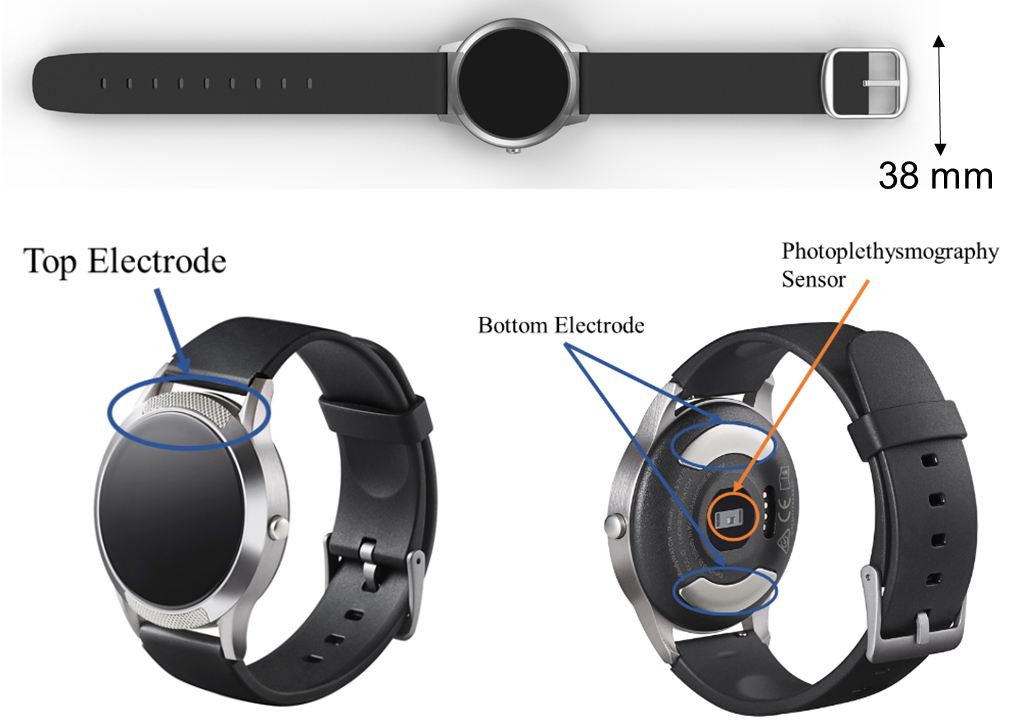
**

**Supplementary Figure S2.** The protocol used in this study for blood pressure (BP) measurements.

**
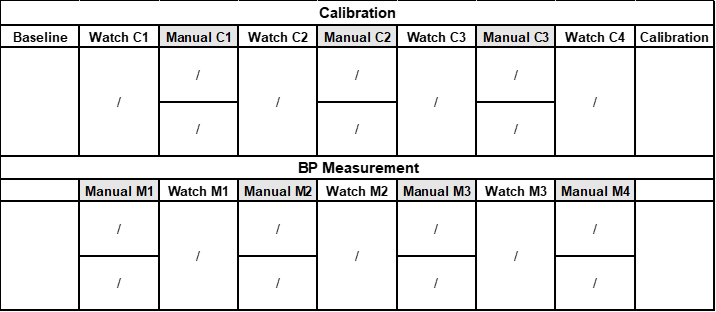
**

**Supplementary Figure S3.** A schematic of the BP estimation algorithm


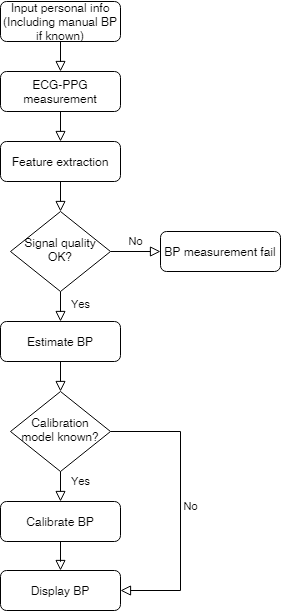


**Supplementary Figure S4.** A schematic of the individual calibration algorithm


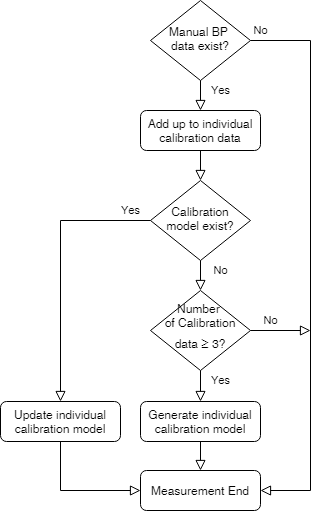

Supplement: Supplementary file 1 — Supplementary Information [file 41598_2020_75892_MOESM1_ESM.docx]
